# The Outcomes at 3 Years in 82 Knees with Kellgren and Lawrence 2–3 Osteoarthritis Treated with an Autologous Protein Fluid Concentrate Produced with a Fluid Volume Reducer

**DOI:** 10.3390/life14101340

**Published:** 2024-10-21

**Authors:** Mitchell Sheinkop, Mary Langhenry, Alaa Abd-Elsayed

**Affiliations:** 1 Cellular Orthopedics, Chicago, IL 60661, USA; ezaca@aol.com (M.S.); mlanghenry@yahoo.com (M.L.); 2 Department of Anesthesiology, University of Wisconsin, Madison, WI 53706, USA

**Keywords:** knee osteoarthritis, autologous protein fluid concentrate, injection therapy

## Abstract

Introduction: Knee osteoarthritis (OA) is a highly prevalent and debilitating condition with significant emotional and economic impacts. Current treatment options may only provide temporary pain relief and are not regenerative, thus the progression of knee OA is not deterred and total knee arthroplasty is inevitable. Injection therapies with orthobiologics possess regenerative potential and are an emerging treatment option. We present a prospective study aimed at examining patients with knee OA who had received an autologous platelet concentrate fluid (APCF) injection produced through a fluid volume reducer. Methods: This was an observational review of the results following an APCF injection in a cohort of patients at a single site. Patients were included in the study if they were diagnosed with K/L grade 2–3 knee OA and treated with an APCF knee injection. Patients were excluded if they had obtained an orthobiologic injection in the three months prior to study enrollment or if baseline data were unavailable. Knee score and function score were used to assess patients at the baseline and post-injection follow-ups. Results: Improvements for knee score were statistically significant for the follow-ups at three months, six months, one year, and three years. Function score improved, with statistically significant changes for the three month and three year follow-ups. Conclusions: Our study demonstrates that there is some utility in using APCF injection for knee OA, with improvements that may be sustained up to three years in some patients.

## 1. Introduction

Osteoarthritis (OA) of the knee is prevalent in 37% of the population over the age of 59 [1]. People with knee OA can be limited in their ability to participate in the activities of daily living, work, and hobbies. In addition, OA and related pain have emotional impacts on patients [2,3]. The economic burden is also substantial, with an estimated $128 billion spent annually in combined direct and indirect expenses [4]. Risk factors for developing OA include increased age, gender (higher prevalence in women), inactivity, obesity, occupational activity, injury to the knee, and varus or valgus misalignment [5,6,7].

To determine the severity or stage of knee OA, MRI and x-ray are used with the Kellgren and Lawrence (K/L) grading system [8]. Absence of any radiographic evidence indicates K/L grade 0, while ambiguous evidence of osteophytes signifies K/L grade 1 and clear evidence of osteophytes signifies K/L grade 2. Definite evidence of joint space narrowing indicates K/L grade 3, and evidence of advanced joint space narrowing where the joint is close to or is bone to bone indicates K/L grade 4. 

The first line treatment for knee OA is topical nonsteroidal anti-inflammatory drugs (NSAIDs) [1]. Other management strategies for knee OA include oral NSAIDs, paracetamol, antidepressants, anti-epileptics, opioids, exercise, weight loss, and physical therapy [9,10]. However, due to the physical and functional limitations experienced by people with knee OA, it may be difficult for them to exercise and lose weight or participate in physical therapy. Additional therapies such as acupuncture, braces, heel wedges, glucosamine, and chondroitin have been trialed, but present insufficient or unclear evidence and are currently not recommended [11]. The aforementioned management strategies should be trialed for all knee OA cases regardless of K/L grade for the purpose of pain relief and improving function. However, these non-surgical treatments may only provide temporary pain relief, as they are not regenerative and will not influence the progression of knee OA (so knee replacement with a prosthesis is inevitable). Total knee arthroplasty (TKA) is a last line treatment that is reserved only for patients who have failed to respond to non-surgical and less invasive treatment options [12]. Commonly, patients who undergo TKA have K/L grade 4 knee OA [13].

The prevalance of knee OA is already high and shows no signs of slowing down [14]. With the older population growing and obesity becoming more common, the number of people with knee OA will only continue to increase [15]. Coupled with the difficulty in obtaining long-term relief with current management regimens, it is imperative that additional treatment options are explored. Since knee OA is a progressive condition that worsens over time, more attention needs to be directed toward therapies that may slow or reverse the progression of the disease. 

Injection therapies with orthobiologics are another option for treating knee OA that have recently gained significant traction [16]. Injections are recommended for K/L grade 2–3 knee OA [17]. These treatment modalities have regenerative potential, because they include bone marrow concentrate, MFat, and platelet-rich plasma (PRP) concentrate as commonly used orthobiologic injectates. Heretofore, the most commonly used injectates for the treatment of knee OA are Hyaluronic Acid (HA) and corticosteroids, although these treatments have come under increasing scrutiny over the years. The American Academy of Orthopaedic Surgeons (AAOS) has placed a “not recommended” tag on HA, and studies of multiple injections of corticosteroids in the same site have shown faster degradation of cartilage. However, a previous large retrospective study found that PRP injections were able to significantly improve pain, function, and quality of life for patients with knee OA [18]. Responder rates were 66.5% and 75.2% at 12 months for patients with K/L grade 3 and 2 knee OA, respectively. Another previous study also reported significant improvements in pain and function in knee OA patients after PRP injections [19]. 

This study also compared MRI images and found significant radiographic evidence which supported the ability of PRP to slow and even reverse the progression of knee OA. We are reporting the outcomes of a prospective study aimed at examining patients with knee OA who received an autologous platelet concentrate fluid (APCF) injection produced through a fluid volume reducer. 

## 2. Materials and Methods

With IRB approval by the Institute of Regenerative and Cellular Medicine (IRCM) (approved 5 July 2018, approval number: IRCM-2018-189), an observational review of the results following an APCF injection at a single site in a cohort of patients who met our inclusion criteria was undertaken by the authors.

### 2.1. Patient Selection

Patients included in the study were diagnosed with K/L grade 2–3 knee OA and scheduled for an APCF knee injection. All patients had imaging before the injection. Patients were required to be between the ages of 18 and 90. All patients also needed to be able and willing to sign Informed Consent and return for scheduled follow-up evaluations. Patients were excluded if they had any type of cortisone, visco-supplementation, PRP, MFAT, or bone marrow aspirate injection in the treated joint within the last three months prior to enrollment in this study. Lastly, patients for whom baseline data were not available were excluded.

### 2.2. Data Analysis

The following patient characteristics were obtained from patient enrollment forms: age, gender, treatment side, concurrent medical history, height, weight, and race. The knee score (i.e., Clinical American Knee Society Score) and function score (i.e., Functional American Knee Society Score) are validated scales that were used to assess patient baseline and post-injection outcomes [20]. 

The knee score uses a clinical examination to assess pain out of 50 points, range of motion out of 25 points, and stability out of 25 points. A total of 100 points on the knee score indicates no pain, adequate range of motion that is greater than 125 degrees, good knee alignment when extended, and no instability. 

The function score evaluates functionality for each patient by assessing walking distance out of 50 points and going up/down stairs out of 50 points. A total of 100 points on the function score signifies being able to walk any distance and up/down stairs normally, all without any support in the form of canes, crutches, or walkers. The knee score and function score were assessed using standard orthopedic scoring methodology. This consisted of subjective pain and function reporting and objective alignment, laxity, and range of motion measurements. The function assessment also included walking, stairs, and the use of assistive aids. The assessment scale is available free of charge at kneesociety.org. 

To assure reproducibility, a single observer (Mary Langhenry, PT, OCs), performed all patient assessments prior to enrollment and at each and every office follow-up via a physical examination incorporating a tape measure and goniometer. Patients were followed up until 3 years after injection.

Statistical analysis was conducted using the SPSS 22 software package. Data were collected on an Excel spreadsheet, then transferred to the SPSS 22 software. Descriptive analysis was performed using numbers and percentages for categorical variables and mean and standard deviation for numeric variables. A paired *t*-test and one-way ANOVA was completed with a *p*-value of ≤0.05 indicating a significant change. A *p*-value of 0.05 as the cut-off for significance is the most commonly used in research, thus we selected itas our cut-off.

### 2.3. Preparation of the Injectate

The ultimate injectate is prepared following a venipuncture allowing for collection of 54 mL of venous blood in a 60 mL syringe containing 6 ccs of Anticoagulant Citrate Dextrose Solution A (ACDA). The 60 ccs of combined fluid is loaded into a PRP, Leukocyte Rich, Concentrating Device and centrifuged at 3200 RPM for 15 min. Next, approximately 30–35 mL of separated plasma is removed from the concentrating device using a 60 mL syringe. A 10 mL syringe is used to remove 4–6 mL of leukocyte-rich buffy coat. The 4–6 mL of buffy coat are combined with approximately 6–8 mL of the separated plasma, totaling 12 mL put through the fluid volume reducer and concentrated to about 2–3 mL of output. A Biomet GPS III kit and Biomet’s Plamax Fluid Volume Reducer were used in an office setting to create the APCF.

### 2.4. Injection Procedure

The patient is positioned supine on the examining table with the knee extended. Careful attention is given to antiseptic skin preparation of the anterior skin surface at the knee to be injected. Using a 3cc syringe and a 27-gauge needle, an injection of 0.5 mL of a local anesthetic is administered to the skin and subcutaneous tissue at the anticipated anterolateral, suprapatellar injection site, with care not to penetrate the knee capsule. Using ultrasound guidance, the joint is evaluated, and a decision is made as to whether to evacuate a synovial effusion prior to injection. Thereafter, an 18-gauge needle is positioned intraarticular under ultrasound control, and the 2–3 mL injectate was administered intraarticular. The needle was quickly withdrawn, and a dressing applied.

## 3. Results

### 3.1. Knee SCORE

A total of 82 patients completed the clinical exam for knee score at baseline and six weeks. Ten patients reported no improvement in their pain. The average age of the patients was 28.9 +/− 6.2 years old and the average BMI was 66.4 +/− 4.3. About 90% of the patients were Caucasians and 54% were females (Table 1). The mean score increased from 68.2 +/− 12.4 to 71.6 +/− 16.8 (*p* = 0.064) (Table 2). Improvements were statistically significant from the three-month to the three-year follow-ups. A total of 77 patients completed the three-month follow-up, where the mean score increased by 9.5 +/− 14.7 points from the baseline (*p* < 0.001). At six months, 62 patients completed the follow-up, and the mean score increased by 6.6 +/− 19.6 points (*p* = 0.01). For 40 patients at the one-year follow-up, the mean score increased by 7.9 +/− 15.7 points (*p* = 0.003). At three years, 14 patients reported a mean increase of 8.6 +/− 12.9 points (*p* = 0.028). The results of the one-way ANOVA indicated a significant difference between the groups (*p* < 0.01). All of the patients who completed the follow-up indicated improvement in their knee score.

### 3.2. Function Score

Initially, 84 patients were assessed for function score at baseline and six weeks, at which time the score of 54.9 +/− 17.5 (+/− 20.9 at six weeks) did not change (*p* = 0.988) (Table 3). For 80 patients at the three-month follow-up, the score increased by a mean of 4.3 +/− 13.5 points (*p* = 0.005). At six months, the score decreased for 64 patients by a mean of 1.1 +/− 13.7 points (*p* = 0.524). For the 44 patients who completed the follow-up at one year, the mean score decreased by 0.3 +/− 14.7 points (*p* = 0.878). The mean score increased by 4.8 +/− 14.2 (*p* = 0.098), 6.8 +/− 10.7 (*p* = 0.033), 4.5 +/− 5 points (*p* = 0.019) at the two- (*n* = 26) and three-year (*n* = 14) follow-ups, respectively. The difference between groups was found to be significant when analyzed using one-way ANOVA (*p* < 0.01). All of the patients who completed the follow up indicated improvements in their function score.

## 4. Discussion

Patients in our study reported, on average, some improvements in pain, stability, and range of motion at all follow-ups, with statistically significant improvements beginning after six weeks and persisting until the three-year follow-up. Improvements in function were reported to be statistically significant at the three-month and three-year follow-ups, with additional improvement at two years. Function score remained unchanged at six weeks and decreased at six months and one year, but these results were not statistically significant. No significant adverse events were reported. While interpreting these results, it is important to acknowledge that the standard deviations were relatively high, indicating that the magnitude of change in knee and function scores varied across patients. 

Our results had both similarities and differences to a previously conducted study. Moretti et al found an 8.4-point increase in knee score and a 10.75-point increase in function score at six months [21]. The improvement of 8.4 was similar to our result and within our 95% confidence interval for the same time period, but the 10.75 increase was not. This difference may be attributed to our study containing patients with higher severity knee OA. Our baseline knee and function scores were 14.4 and 28.07 points lower compared to this previous study. There are also several previous studies that are not directly comparable to our results due to the use of different assessment scales, but these studies all demonstrate the potential benefit in a PRP APCF injectate for knee OA [22,23,24,25,26,27]. 

Finding an effective treatment for knee OA that influences the disease progression will help reduce the number of patients who need TKA. Without such a therapy, it is estimated that by 2030, the population that needs TKA will increase by 673% [28]. Revision TKA surgery is projected to increase by 601% during the same time and will only continue to rise as more patients undergo TKA. Furthermore, an effective knee OA management strategy will improve the safety and well-being of patients’ day-to-day life. Globally, falls account for the second largest number of unintentional deaths due to injury [29]. Fractures and subdural hematomas are potential injuries that can occur following a fall [30]. Knee pain as a result of OA has been reported to be associated with a greater risk of falls and hip fractures, thus increasing mortality and morbidity [31]. This increased risk may be due to the reduction in muscle strength, increased stiffness, and worsening in balance experienced by those who suffer from knee OA [32,33]. In addition to the risk of falls and fractures in patients with OA, the risk of developing cardiovascular disease is also greater [34]. This increase in risk may be attributed to OA limiting a patient’s ability to participate in physical activities and exercises.

The mechanism by which APCF injections implement their effects is one of promoting wound healing and tissue repair [35]. APCF comprises high concentrations of platelets, which could help aid in longer term outcomes [36], leukocytes for the promotion of IL-1RA [37], and concentrated plasma-poor proteins, which contain a myriad of growth factors and proteins [38,39,40]. Important growth factors involved in the healing and repair process, such as insulin-like growth factor-1 (IGF-1), white blood cells (WBCs), vascular endothelial growth factor (VEGF), transforming growth factor beta (TGF-β), and platelet-derived growth factor (PDGF) modulate cell migration, proliferation, and differentiation [37,40]. Additionally, the upregulation of extracellular matrix synthesis and angiogenesis occurring in APCF could result in higher yields of interleukin-1ra, soluble TNF, and Alpha 2 Macrogloblin, helping to provide longer term relief [41,42].

Our study is unique in that we used a novel injectate for the treatment of knee OA. This is the also the first study to the authors’ knowledge in which a fluid volume reducer was used to obtain the final injectate amount. This allowed us to inject a small volume of APCF that was consistent across all of the study participants. Additionally, the exclusion of patients who had used other orthobiologic injection therapies in the previous three months ensured that the outcomes from this study were solely due to the APCF injectate. A limitation for our study was the loss of follow-up for some patients. Despite the loss of follow-up, our study still provides long term data—up to three years—that may influence future studies. Our study was also limited by an inability to include serial imaging follow-up. Another limitation was out of pocket expenses that averaged $3000 per patient, which may have been a barrier for potential study participants. Future studies should implement radiographic imaging to assess whether any change in the degeneration of cartilage and synovium was observed or whether any regeneration occurred. Without imaging, it is not possible to determine for certain whether the injectate has regenerative potential or plays any role in slowing the procession of knee OA. The lack of a control group was a limitation as well. 

## 5. Conclusions

Our study demonstrates that there is some utility in using APCF injections for knee OA. Improvements following APCF injections may be sustained for up to three years in some patients.

## Figures and Tables

**Table 1:** Patients’ demographics.

Category		
Age	28.9 +/− 6.2 years old	
BMI (Body mass index)	66.4 +/− 4.3	
Gender	54% females	46% males
Race	90% Caucasians	10% others

**Table 2:** Knee Score.

	Mean (SD)	Mean Difference from Baseline (SD) [95% Confidence Interval]	*p*-Value *	N
Baseline	68.2 (12.4)	−3.4 (16.5) [−7 to 0.2]	0.064	82
6 weeks	71.6 (16.8)			
Baseline	69.2 (13.2)	−9.5 (14.7) [−12.8 to −6.1]	<0.001	77
3 months	78.7 (12.3)			
Baseline	71.4 (12.9)	−6.6 (19.6) [−11.6 to −1.6]	0.01	62
6 months	78 (16.4)			
Baseline	70.4 (12.9)	−7.9 (15.7) [−12.9 to −2.8]	0.003	40
1 year	78.3 (14.9)			
Baseline	66.6 (14.8)	−8.6 (12.9) [−16 to −1.1]	0.028	14
3 years	75.1 (11.8)			

* from paired *t*-test.

**Table 3:** Function Score.

	Mean (SD)	Mean Difference from Baseline (SD) [95% Confidence Interval]	*p*-Value *	N
Baseline	54.9 (17.5)	0 (14) [−3.1 to 3]	0.988	84
6 weeks	54.9 (20.9)			
Baseline	61.7 (21.1)	−4.3 (13.5) [−7.3 to −1.3]	0.005	80
3 months	66 (19.9)			
Baseline	63.7 (23.2)	1.1 (13.7) [−2.3 to 4.5]	0.524	64
6 months	62.6 (21.1)			
Baseline	60 (21)	0.3 (14.7) [−4.1 to 4.8]	0.878	44
1 year	59.7 (25.2)			
Baseline	58.5 (24.4)	−4.8 (14.2) [−10.6 to 0.9]	0.098	26
2 years	63.3 (32.8)			
Baseline	64.3 (14.5)	−6.8 (10.7) [−12.9 to −0.6]	0.033	14
3 years	71.1 (20.2)			

* from paired *t*-test.

## Data Availability

Data is available upon request to the corresponding author.
